# Is Peri-Implant Probing Causing Over-Diagnosis and Over-Treatment of Dental Implants?

**DOI:** 10.3390/jcm8081123

**Published:** 2019-07-29

**Authors:** Pierluigi Coli, Lars Sennerby

**Affiliations:** 1 Edinburgh Dental Specialists, Edinburgh EH2 4BA, UK; 2 Department of Maxillofacial Surgery, University of Gothenburg, 413 90 Gothenburg, Sweden

**Keywords:** dental implants, mucositis, peri-implantitis, diagnosis, over-treatment, iatrogenic damage

## Abstract

Pocket probing depth (PPD) and bleeding on probing (BOP) measurements are useful indices for the assessment of periodontal conditions. The same periodontal indices are commonly recommended to evaluate the dental implant/tissue interface to identify sites with mucositis and peri-implantitis, which, if not treated, are anticipated to lead to implant failure. The aim of the present narrative review is to discuss the available literature on the effectiveness of probing at dental implants for identification of peri-implant pathology. There is substantial clinical evidence that PPD and BOP measurements are very poor indices of peri-implant tissue conditions and are questionable surrogate endpoints for implant failure. On the contrary, the literature suggests that frequent disturbance of the soft tissue barrier at implants may instead induce inflammation and bone resorption. Moreover, over-diagnosis and subsequent unnecessary treatment may lead to iatrogenic damage to the implant-tissue interface. Despite this, the recommendations from recent consensus meetings are still promoting the use of probing at dental implants. For evaluation of implants, for instance at annual check-ups, the present authors recommend a clinical examination that includes (i) a visual inspection of the peri-implant tissues for the assessment of oral hygiene and the detection of potential redness, swelling, (ii) palpation of the peri-implant tissues for assessment of the potential presence of swelling, bleeding, suppuration. In addition, (iii) radiography is recommended for the assessment of crestal bone level for comparison with previous radiographs to evaluate potential progressive bone loss even if there is a need for more scientific evidence of the true value of the first two clinical testing modes.

## 1. Introduction

The ultimate goals of the maintenance phase of implant treatment are to preserve the function and the aesthetics of the rehabilitation as well as the stability /health of the peri-implant tissues for as long as possible. From a research point of view, the goal is to monitor the treatment outcomes: implant survival and success/failure. One evident problem is that the definitions of survival, success/failure used in more recent publications do not necessarily reflect the patient/clinician perceived successful maintenance of the function and aesthetics of the treatment. In other words, the definitions of disease, which would motivate a clinical intervention to cure the disease, do not seem to reflect the clinical reality. Thus, the first questions to be answered are “what should be considered pathology at a dental implant?” and “what diagnostic tools are available?” With regards to the natural dentition, there has been a wide agreement within the scientific community in relation to disease definitions and available diagnostic tools [1]. Despite the recent World Consensus meeting in Chicago [1], a similar consensus with regards to definition of pathologies and available diagnostic tools does not seem to exist for peri-implant tissue conditions [2,3,4,5]. The controversy regarding the application of periodontal indices to dental implants is not a recent one [6]. The basis for the disagreement can be found in the difference in the viewing of the peri-implant tissues. Can similar disease definitions and diagnostic tools be used in natural dentition and in situations where the natural dentition has been replaced with implant-retained restorations? The question is logical since there are no doubts that the periodontium and the peri-implant tissues ought to be regarded as two very different entities [5,7,8].

Is it correct to assume that similar reactions to infection, occlusal trauma, trauma from probing can be expected for the periodontium and the peri-implant tissues? Is there a similar pattern of disease progression? And if these aspects differ between the two entities, is it correct to use similar diagnostic tools? There is evidence in the literature that the peri-implant tissues are more susceptible to inflammatory reactions, a phenomenon also confirmed immunohistochemically with increase of inflammatory infiltrate in comparison to teeth [9] that lesions around implants and teeth have critical histopathologic differences [10], and that there are differences in the onset and progression of the periodontal and peri-implant diseases [11]. Becker and co-workers examined the differences and similarities between peri-implantitis and periodontitis underlying disease mechanisms [12]. On the basis of quantitative transcriptome analysis, peri-implantitis and periodontitis exhibited significantly different mRNA signatures, supporting the hypothesis of peri-implantitis being a complex inflammatory disorder with a unique pathophysiology. While in peri-implantitis tissue, the regulation of transcripts related to innate immune responses and defense responses were dominating, in periodontitis tissues, bacterial response systems prevailed [12]. Several authors questioned the role of infection as a principal cause of peri-implant diseases. For instance, Koka and Zarb questioned whether peri-implantitis is a disease entity at all. The Authors refuted the bacterial implications and suggested terms such as osseoinsufficiency or osseoseparation to describe problematic implants [13]. Moreover, in a review article, Qian and co-workers failed to find evidence that primary infection causes marginal bone resorption around implants [14]. Osseointegration has been suggested to represent a foreign body reaction to biomaterials and its long-term clinical function depending on a foreign body equilibrium that, if disturbed, may lead to impaired clinical function of the implant [15]. Recently, dental implants have been suggested having more in common to orthopaedic implants in terms of foreign body reaction and failure pattern than to teeth [16]. Given these fundamental differences between the periodontal and peri-implant tissues, can periodontal indices be used as reliable diagnostic tools for peri-implant tissues?

## 2. The Risk of Using Surrogate Endpoints for Prediction of Fatal Events

In the most recent definition and diagnosis requirements for peri-mucositis and peri-implantitis, the use of probing around dental implants has been recommended for the detection of presence of bleeding on probing (BOP), of increases in pocket probing depth (PPD) or of presence of PPD equal or more than 6 mm [17]. Thus, periodontal indices are recommended as biologic measures to distinguish between health and disease conditions and as surrogate endpoints as substitutes for fatal events (implant failures) in clinical trial designs. The underlying assumption is that an improvement in the surrogates would benefit the patient and that it is equivalent to reducing the rate of implant losses [18]. Whenever surrogate endpoints (or biological markers) are used, the link between the surrogate and the true clinical event is critical since the use of non-validated endpoints may be more harmful than beneficial. A classic example in the field of medicine is the clinical investigation carried out on the assumption that arrhythmia is a risk factor for acute myocardial infarction and that, as a consequence, the suppression of arrhythmia would reduce the risk of death by infarction. The investigation had to be stopped when it became clear that the antiarrhythmic agents successfully reduced the number of arrhythmia episodes but increased the risk of dying by infarction. Clearly, the reduction of arrhythmia was not a good surrogate of treatment benefit to the patient [19]. One can question whether the high prevalence of periodontal diseases claimed in more recent years and the alarming possibility of peri-implantitis becoming a major future health problem [20] is correct or whether it is based on the wrong choice of surrogate endpoints (biologic markers), and therefore cause unnecessary alarmism and overtreatments which are of no benefit to the patients.

## 3. The Use of Periodontal Indices to Diagnose Peri-Implant Disease

A systematic review of peri-implantitis therapy published in 2010 showed that PPD, BOP and clinical attachment level (CAL) were the most frequently reported surrogate markers for important clinical events such as implant failure, despite the fact that these surrogate markers/endpoints had not yet been validated [18]. Has anything changed in the last nine years? Has the use of periodontal indices around dental implants been validated since then? Periodontal probing has been a common basic diagnostic tool around teeth since investigations in the 1970s demonstrated that PPD measurements around teeth provide information regarding the ability of the periodontium to withstand probe penetration as a measure of the inflammatory conditions of the tissues. In case of an inflamed periodontium, the tip of the probe penetrates the epithelium into the connective tissue, overestimating the depth of the histological pocket. In case of a healthy gingiva, the tip of the probe fails to reach the most apical cell of the epithelium due to the increased resistance of the periodontal tissues, thus underestimating the depth of the histological pocket [21,22,23]. Ericsson and Lindhe confirmed this finding in animals but also showed that in healthy soft tissues conditions, probe penetration was more advanced at implants than at teeth and concluded that the differences between the attachment structure of teeth versus peri-implant mucosa makes the conditions for PPD measurements at teeth and implants different [24]. In contrast, Lang and co-workers reported that in healthy and mucositis sites, the probe tip was located at the most apical cell of the junctional epithelium, whereas in the case of ligature-induced peri-implantitis sites, the probe tip penetrated into the connective tissue. They concluded that probing around implants represents a good technique for assessing the status of peri-implant mucosal health or disease. The difference in results between the two studies was attributed to the lower probing forces in the last study, which was 0.2 N versus 0.5 N [25].

More relevantly, on an evidence-based scale, investigations in humans failed to detect a positive correlation between the presence of periodontal signs of inflammation (PPD, BOP) and peri-implant bone loss. In a cross-sectional study by Lekholm and co-workers with a mean follow-up time of 7.6 years of 125 implants placed in 20 partially edentulous patients reported that 60% of the pockets measured more than 4 mm and that 80% of the sites were positive for BOP despite a limited mean bone loss of 0.07 mm annually. The microflora was periodontally non-pathogenic in nature in 94% of the samples and soft tissue biopsies showed a healthy mucosa in 95% of the cases. Thus, bleeding of the peri-implant tissues and deep pockets had no correlation to crestal bone loss or to the presence of a pathogenic microflora or to histological changes indicative for signs of periodontitis [26]. These findings were confirmed several years later by Dierens and co-workers, who reported of lack of correlations between PPD or BOP and crestal bone loss around single implants functional for 16–22 years. PPD and BOP were found to be of poor diagnostic value [27]. Recently also Winitsky and co-workers confirmed these observations, reporting of a lack of correlation between radiographically-detected crestal bone loss and periodontal indices such as PPD >6 mm and BOP in a 14–20 follow-up of 48 single anterior maxillary implants with a survival rate of 96%. The authors concluded by raising the question of whether PPD and BOP should be used as diagnostic measurements of implant health [28].

## 4. Evidences That Pocket Probing Depth Is a Poor Indicator of Ongoing Peri-Implant Pathology

The thickness of the healthy soft tissues surrounding implants has been reported to range between 1.85 and 5.75 mm in beagle dogs [29]. A clinical study showed values ranging from of 0.85 to 6.85 mm, but even papillae of 7–9 mm were observed [30]. Often, deeper PPDs are found at implant sites inserted in partially edentulous ridges compared to edentulous ridges [31]. Kan and co-workers reported the interproximal thickness of healthy peri-implant mucosa to be roughly 6 mm (SD 1.2) in maxillary anterior single implant with a mean functional time of 3 years [32]. Long-term clinical investigations have clearly shown that the probing depth of healthy peri-implant mucosa is often more than 4 mm (60% to 63%) [26,28,33] and up and over 6 mm (15% to 23%) [27,28] and that successful implants with over 18 years of function might have a history of PPD up to 9 mm [28,33]. From animal and human studies, it is evident that PPD depends on the thickness of the soft tissue at the time of implant placement and on the anatomical circumstances. For these reasons, there is no specific pocket depth that can indicate disease conditions. This fact is acknowledged in the new classification scheme for periodontal and peri-implant diseases and conditions, where in the peri-implant health definition it is stated that “it is not possible to define a range of probing depths compatible with peri-implant health” [1], and it is reflected in the fact that for the diagnosis of peri-implant health, it is mentioned that probing depths depend on the height of the soft tissue at the location of the implant [17]. Unfortunately, in the same paper, it is stated that in the absence of previous examination data, diagnosis of peri-implantitis requires the presence of bleeding and/or suppuration on gentle probing, probing depths ≥6 mm, and bone levels ≥3 mm apical of the most coronal portion of the intraosseous part of the implant.

Obviously, in light of what was discussed so far, the recommendation of using a 6-mm deep pocket (which appears to be an arbitrary choice) as one of the indicators of peri-implantitis appears to be highly questionable.

In the same paper, it is stated that for the diagnosis of peri-implantitis among other parameters, “an increased probing depth compared to previous examinations” is required.

The use of changes in PPD to establish a diagnosis of peri-implantitis has been questioned [5]. Schou and co-workers have shown in animal studies that PPD assessments could not distinguish between peri-implant sites with or without crestal bone loss and that the only correct indication of bone level stability was obtained by radiographs [34]. Furthermore, even mild inflammation was associated with deeper probe penetration around implants in comparison to teeth with no correlation to presence of bone loss [35]. In a more relevant 5-year clinical prospective investigation, Weber and co-workers assessed the ability of several clinical parameters (Suppuration, PI. BOP, PPD, PAL, mobility) to predict crestal bone loss as detected on radiographs in 112 ITI implants [36]. The authors reported no bone changes between years one and five in comparison to increasing PPD values during the five years. The cumulative predictive power of the six clinical parameters with regards to bone loss was reported to range from 2.8% to 14.3%. It was concluded that “the low levels of correlation between the individual and cumulative clinical parameters with radiographically measured bone loss, suggests that these measures are of limited clinical value in assessing and predicting future peri-implant bone loss”. Healthy peri-implant soft tissues have been reported in sites with increased PPD values by Giannopoulou and co-workers in a 9-year follow-up of 61 maxillary anterior implants using clinical, microbiologic and biochemical parameters, thus showing a very poor correlation between PPD changes and the presence of pathology at the peri-implant tissues [37]. From the above discussed data, it appears that an increase in probing depth around implants does not necessarily mean that loss of bone or clinical attachment has occurred. Since the key parameter for establishing a diagnosis of peri-implantitis is bone loss and since the latter cannot be properly identified by a specific, pre-established PPD value or by changes in PPD, the use of probing for PPD assessments around implants does not appear to be a validated diagnostic tool.

## 5. Evidences That Bleeding on Probing Is a Poor Indicator of Ongoing Peri-Implant Pathology

For the detection of pathology and consequent treatment needs, BOP is a key parameter, according to the most recent recommendations, since BOP is used as a diagnostic parameter for the detection of both peri-mucositis and peri-implantitis [17]. This recommendation assumes that healthy peri-implant soft tissues do not test positive to BOP, whereas only diseased sites do (or, at least, that there is a statistically significant clinical difference between healthy and diseased sites when it comes to BOP positivity test). While Lang and co-workers’ Beagle dog investigation detected constant increases in BOP from healthy peri-implant sites to ligature-induced peri-mucositis sites to ligature-induced peri-implantitis [25], Ericsson and Lindhe’s Beagle dog investigation reported of the presence of BOP for the majority of the healthy peri-implant sites [24]. One investigation comparing teeth and implants with respect to soft tissue healing revealed that peri-implant healing as determined by crevicular molecular composition differs from periodontal healing and suggested that peri-implant tissues represent a higher pro-inflammatory state [38]. Thus, the peri-implant soft tissues could be considered to be in a state of subclinical chronic inflammation. In fact, cross-sectional studies have shown that BOP can be detected at the majority of sites, showing stable peri-implant tissues.

With a definition of peri-implantitis as an association of BOP and any bone-level alterations at implant sites occurring between the 1-year and the 5-year (up to 23 years) follow-up examinations, Fransson and co-workers reported the presence of BOP in 93.9% of the 197 implants with “progressive” bone loss and in 90.9% of the 285 implants with stable bone level [39].

Roos-Jansåker et al reported that peri-implant mucositis, diagnosed by BOP, was detected in approximately 70% of functioning implants after 9 to 14 years. The prevalence of peri-implantitis, defined as bone loss of at least 1.8 mm following the first year of function, combined with BOP and/or pus, was 16% at patient level and 6.6% at implant level. Interestingly, 42.2% of the implants had stable bone levels and still showed BOP or suppuration, and 8.4% of the implants showed bone level gain even in the presence of BOP or suppuration [40]. A very poor correlation between presence of BOP and presence of peri-implant diseases has been reported in several long-term clinical studies. Lekholm at al reported the BOP of 80% around implants, showing an annual bone loss of 0.07 mm and a 95% healthy mucosa at biopsies [26]. Dierens and coworkers reported 81% BOP around implants, showing stable conditions for 16 to 22 years of function [27]. Winitsky and co-workers reported 71% BOP around implants, showing stable conditions for 14 to 20 years of function [28]. French and co-workers, in a large cohort study including 4591 Straumann implants from 2060 subjects evaluated up to ten-year follow-up, reported that BOP was a common finding, detected in more than 40% of the implants during the study. Despite the high prevalence of bleeding, less than 3% of implants exhibited more than 1 mm crestal bone. The presented data indicated that minimal bleeding did not correlate with bone loss, whereas profuse bleeding or suppuration did [41].

The type of implant, two-piece vs. one-piece, may affect the soft tissue bleeding response to probing. The presence of a chronic infiltrate at the implant-abutment interface of two-piece implants has been reported and has been attributed to the microgap between the implant and the abutment [42,43]. In contrast, the connective tissue surrounding one-piece implants has been reported to be inflammation-free, possibly due to the absence of a microgap [44]. This could partially explain the high BOP prevalence detected at stable peri-implant sites in investigations on two-piece implants [26,27,28,39,40] and the lower percentage of BOP (40%) detected at stable peri-implant sites around one-piece implants. In case of one-piece implants, the presence of profuse BOP had a higher correlation to crestal bone loss compared to the poor correlation of minimal BOP [41].

The type of abutment material has been demonstrated to have an effect on BOP values in a recent systematic review, where increased BOP values over time were demonstrated for Ti when compared to Zi abutments [45]. Implant position (anterior v posterior), gender and PPD have been shown to be factors affecting the probability of a peri-implant site to be positive to BOP. In 112 patients, data related to 1725 peri-implant sites showed that the probability to bleed on probing increases for implants placed in anterior compared to posterior areas of the dentition, for implants placed in female patients compared to male patients, and that for each mm increase in PPD, there is a corresponding 10% increase in the probability of detecting BOP at the site [46].

Thus, BOP seems to depend on the implant type (two-piece/one-piece), the type of abutment material, the implant position (anterior/posterior), the patient’s gender and the PPD of the probed site without correlation to disease presence.

It has been shown that BOP can be detected in the majority of healthy peri-implant sites and cannot therefore be reasonably used to distinguish between peri-implant health and disease.

The investigations that attempted to establish the validity of the use of BOP as a predictor of future crestal bone loss around implants have failed to produce convincing result to justify the use of BOP as an appropriate diagnostic test. Jepsen and co-workers reported no difference in BOP between sites with progressive peri-implant PAL loss (rather than progressive bone loss) or stable sites. The authors pointed out that probing might provoke a nonspecific bleeding that is unrelated to the amount of inflammation. Thus, BOP as a diagnostic test for progressive PAL loss had a sensitivity of 70% and a specificity of 32%. In other words, BOP was of limited value in the implant-specific diagnosis when examined as positive predictors for peri-implant attachment loss. However, BOP demonstrated a higher negative predictive value and it was concluded that negative scores can serve as indicators of stable peri-implant conditions [47]. Monje and co-workers found that the diagnostic accuracy of BOP was not enough to distinguish healthy from peri-implantitis sites (defined as presence of inflammation and 2 mm crestal bone loss). A visual sign, such as mucosa redness, was reported having a much better diagnostic accuracy in monitoring the presence of pathology. For the clinical parameters investigated (PPD, BPO, mucosa redness, PI), it was found that, as diagnostic tests, their specificity surpasses their sensitivity in the detection of peri-implant diseases. The authors therefore concluded that the diagnosis of peri-implant diseases cannot rely on a single clinical parameter but rather requires a combination. More interestingly, it was pointed out that progressive radiographic bone loss must be cautiously examined to reach definitive diagnosis and avoid overtreatment [48]. Weber and co-workers reported that the cumulative predictive power of six clinical parameters (Suppuration, PI. BOP, PPD, PAL) with regards to bone loss ranges from 2.8% to 14.3% and concluded that “these measures are of limited clinical value in assessing and predicting future peri-implant bone loss” [36]. A recent systematic review and meta-analysis demonstrated that for BOP-positive implants, there was a 24.1% chance of being diagnosed with peri-implantitis, while for BOP-positive patients, there was a 33.8% probability of being diagnosed with peri-implantitis. It was concluded that clinicians should be aware of the considerable false-positive rate of BOP to diagnose peri-implantitis [49].

## 6. Mismatch between Known Clinical Facts and Recommendations from Consensus Meetings

It is interesting to note that review articles [5,49,50] and prospective studies [36,48] conclude that periodontal indices are unreliable tools for examining implants. Yet, the consensus meetings that often commission the reports keep recommending the use of periodontal indices around implants.

As discussed by Coli and co-workers, the efficacy of a diagnostic test is affected by the prevalence of the disease in the investigated population [5]. With increases in the disease prevalence, the probability that a person with a positive test result does in fact have the disease increases. Thus, two factors are of importance in order to properly establish an accurate disease diagnosis and avoid high figures of false positives. The first factor is the availability of a diagnostic test with high sensitivity and specificity. This has not yet been proven to be the case for any of the periodontal indices usually applied around dental implants. The second factor is the application of the diagnostic test to a population that has a high prevalence of the disease. Studies using the presence of BOP and a pre-established amount of crestal bone loss can result in high prevalence of peri-mucositis and peri-implantitis, however, if more stringent values of crestal bone loss are applied, much lower prevalence values are presented [50].

Long-term clinical investigations on machined implants are showing that despite the presence of several clinical parameters that would be considered indicative of pathology in the case of natural dentition (BOP, increases in PPD, suppuration), peri-implant tissue conditions were generally stable for over 18 years with only 2.5%–5% of implants showing progressive bone loss [27,28,33,51,52]. A review including ten different publications on three brands of moderately rough surfaces with ten- year or longer follow-up times reported a 2.7% peri-implantitis prevalence [7]. Jemt and co-workers reported that the incidence of surgery related to peri-implantitis problems carried out at the Branemark clinic was on an average 1.2% of followed-up patients per year (on an average, 1294 patients per year) during an 8-years period [53]. Thus, long-term clinical studies on machined as well as on modern micro-rough implant surfaces are indicative of a low 1.2%–5% prevalence of peri-implantitis and implant losses due to peri-implantitis. With such low peri-implantitis prevalence figures and with clinical parameters with poor accuracy as diagnostic tests, the probability of a dental implant being correctly diagnosed as suffering from peri-implant diseases appears to be very low and the risk of overtreatment very high.

Two investigations highlight the poor accuracy of periodontal indices causing overdiagnosis in several cases and failing to correctly identify implants that will suffer crestal bone loss in the future. In a follow-up study based on the population described by Fransson and co-workers [39,54], Jemt and co-workers showed that 9 years after the initial diagnosis of peri-implantitis, 31% of the patients presented with implants with bone loss >2mm/year or with implant failures, whereas 69% of them showed no problems with their implants [55]. A total of 91.4% of the implants in the peri-implantitis diagnosed patients showed no or smaller annual bone loss than <0.2mm during the 9 years from the diagnosis. The authors reported a low prevalence of obvious bone loss at implants (>0.2 mm/year) with a comparable distribution between “affected” and “not affected” implants. Hence, the definition of peri-implantitis used in the Fransson and co-workers study [39], bone loss associated with BOP, was shown to be a poor predictor of future bone loss and implant failure and, consequently, a poor indicator of treatment needs. In a follow-up study based on the population described by Roos-Jansåker and co-workers in 2006 [40], Renvert and co-workers reported that 12 years after the initial diagnosis of peri-implantitis and surgical treatment, 23% of the patients presented with implants with further bone loss ≥3 threads. In the remaining 77% of the subjects, bone gains (15%) or no further bone loss or bone loss <3 threads were detected [56]. Out of the subjects that at the first examination did not have peri-implantitis, 15% were diagnosed as having at least one implant with bone loss of ≥3 threads in the 21–26-year examination. For the 9–14 years examination, 58% of the individuals had been diagnosed with mucositis. Of those, 14% were found to have developed peri-implantitis at the 21–26-year examination. On the other hand, 22% of the patients without any sign of mucositis after 9–14 years had developed peri-implantitis at a later stage. Thus, a diagnosis of mucositis established after 9–14 years was not predictive for development of peri-implantitis after 21–26 years, nor was the diagnosis of peri-implantitis after 9–14 years predictive of further bone loss at 21–26 years. It seems evident that the use of a dichotomous diagnostic criterion (bleeding yes or no) for the definition of peri-mucositis and the arbitrary choice of a defined bone loss in association with BOP (surrogate endpoints) for the definition of peri-implantitis, does not capture the long-term true outcome (endpoint) in the form of implant failure and could result in massive overtreatment of implant patients. In fact, patients treated by oral hygienists and/or had experienced peri-implantitis surgery did not seem to show any more favourable progression of bone loss as compared with non-treated patients [55,56].

## 7. The Risk of Iatrogenic Damage by Probing of Dental Implants

One important aspect that has not been debated in the literature and that has not yet been properly tested is the fact that probing around implants could potentially result in trauma to the peri-implant soft tissues with consequent inflammation, apical proliferation of the epithelium and consequent bone loss. There is strong evidence in the literature that the mechanical disruption of the mucosal barrier around an implant should be considered as a connective tissue wound resulting in epithelial proliferation to cover the wound and in bone resorption to allow a connective tissue barrier of proper dimensions to reform in order to re-establish a “biological width”. Repeated abutment dis/reconnections with a consequent disruption of the peri-implant soft tissue barrier have been shown to cause crestal bone resorption around dental implants in animal studies [57,58] and in short-term and long-term clinical investigations, as confirmed in several meta-analysis reports [59,60,61]. Although this limited crestal bone resorption does not seem to be clinically relevant, this established fact should at least raise the doubt that regular peri-implant tissue probing assessments might repeatedly disrupt the soft tissue barrier with consequent serious iatrogenic effects on the stability of the peri-implant tissues in the long term.

Another serious aspect to be considered is the overdiagnosis and overtreatment caused by the use of periodontal indices. In periodontology, it is well established that the presence of BOP is not an indicator of future periodontal tissue loss, but rather that the absence of BOP is a good predictor of periodontal stability [62]. For this reason, during the active and maintenance phases of periodontal treatment, 4-mm-deep or deeper sites showing BOP are treated by scaling and root planing. This zero-tolerance approach certainly results in overtreatment in several cases but does not result in damages to the periodontal tissues and is therefore accepted and recommended. The same approach in the case of dental implants seems to be unjustified and potentially dangerous. As discussed above, there is no evidence in the literature that the presence of BOP at an implant site is a sign of pathology (peri-mucositis or peri-implantitis) with a consequent treatment needed. The zero-tolerance approach to bleeding in the case of dental implants could not only result in overtreatment, but, in fact, in the triggering and the establishment of a difficult-to-manage inflammation in the soft tissues and excruciate into a foreign-body reaction.

Different techniques are used to achieve decontamination of the abutment/implant surfaces. Calculus is removed by manual debridement, such as conventional or ultrasonic scaling, resulting in the release of Ti particles in the surrounding tissues and in surface changes affecting the corrosion resistance of the material [63,64,65,66,67]. Orthopaedic studies have shown that the presence of titanium particles from wear of limb prosthesis could over-express pro-inflammatory cytokines, that are related to the osteolysis process, culminating in bone loss around the implant and prosthesis failure [68]. A recent review concluded that Ti particles and corrosion products from dental implants can have adverse effects on biological tissue [69]. Titanium particles released by ultrasonic scaling on dental implants have been shown to activate inflammatory responses in in vitro studies: activating the DNA damage response pathway in oral epithelial cells [70] or resulting in an increased secretion of IL-1β, IL-6, and TNF-α in cultured human macrophages [71,72,73], inducing bone resorption [71]. In vivo, titanium particles have been found in soft and hard tissue biopsies retrieved from sites with peri-implantitis [74,75]. Peri-implantitis tissues have been shown to contain high concentrations of Ti compared to controls from periodontitis tissues, leading to the conclusion that the high Ti content in peri-implant mucosa has the potential to aggravate inflammation [76]. Furthermore, greater levels of dissolved titanium have been detected in submucosal plaque around implants with peri-implantitis compared with healthy implants, indicating an association between titanium dissolution and peri-implantitis [77].

Since Ti particles can be released from surfaces of dental implants because of mechanical wear and because of contact to chemical agents and/or with substances produced by adherent biofilm and inflammatory cells, Mombelli and co-workers suggested that rather than being the trigger of disease, the observed higher concentration of Ti particles in inflamed peri-implant tissues could be the consequence of the presence of biofilms and inflammation [78]. However, in a recent animal model, it was shown that Ti particles induce an inflammatory response with consequent bone loss and that both inflammation and bone loss can be inhibited by the use of blockers targeting specific inflammatory cytokines. The specific role of inflammatory cytokines in the development of Ti particle-induced peri-implantitis was therefore clearly demonstrated [79]. Another investigation using a different animal model further confirms that Ti particles can induce inflammatory bone loss even in the absence of a bacteria infection and that the inflammatory response can be inhibited by blocking macrophage activity [80]. Thus, there is increasing evidence that dental implant degradation products released by corrosion and/or abrasion during mechanical debridement can act as foreign bodies, initiating the release of inflammatory mediators associated with bone resorption, as already described in the case of orthopaedic implants [81]. Hence, non-surgical implant debridement, incorrectly triggered by the detection of BOP at one otherwise healthy and stable implant site, could result in alterations of the implant surface, with the release of Ti particles (at the time of debridement and/or as a later consequence of the surface corrosion) and initiation of a foreign-body reaction.

## 8. Conclusions and Recommendations Regarding Evaluation of the Implant-Tissue Interface

Periodontal indices do not seem to be reliable indicators for appropriate diagnosis and treatment needs around dental implants. Apparently, they do not provide better information than visual inspection and detection of mucosa redness. Probing around dental implant is more uncomfortable for the patient compared to probing around teeth. Probing around implants could potentially create a trauma in the peri-implant scar tissue that could become difficult to manage. All the information gathered from probing (BOP, PPD, CAL) needs to be associated to the radiographic assessment of crestal bone levels to establish a definitive diagnosis and avoid overtreatment. Therefore, it appears to be more logical to avoid any risks of disturbing the peri-implant tissues with probing and instead proceeding with a clinical examination that includes (1) a visual inspection of the peri-implant tissues for the assessment of oral hygiene and the detection of potential redness, swelling, (2) palpation of the peri-implant tissues for assessment of the potential presence of swelling, bleeding, and suppuration, and (3) radiography for the assessment of crestal bone level for comparison with previous radiographs to evaluate potential progressive bone loss even if there is a need for more scientific evidence of the true value of the first two clinical testing modes.

## References

[1] Caton J.G., Armitage G., Berglundh T., Chapple I.L.C., Jepsen S., Kornman K.S., Mealey B.L., Papapanou P.N., Sanz M., Tonetti M.S. (2018). A new classification scheme for periodontal and peri-implant diseases and conditions—Introduction and key changes from the 1999 classification. J. Clin. Periodontol..

[2] Tomasi C., Derks J. (2012). Clinical research of peri-implant diseases—Quality of reporting, case definitions and methods to study incidence, prevalence and risk factors of peri-implant diseases. J. Clin. Periodontol..

[3] Derks J., Tomasi C. (2015). Peri-implant health and disease. A systematic review of current epidemiology. J. Clin. Periodontol..

[4] Albrektsson T., Chrcanovic B., Ostman P.O., Sennerby L. (2017). Initial and long-term crestal bone responses to modern dental implants. Periodontology 2000.

[5] Coli P., Christiaens V., Sennerby L., Bruyn H. (2017). Reliability of periodontal diagnostic tools for monitoring peri-implant health and disease. Periodontology 2000.

[6] Brånemark P.I., Hansson B.O., Adell R., Breine U., Lindström J., Hallén O., Ohman A. (1977). Osseointegrated implants in the treatment of the edentulous jaw. Experience from a 10-year period. Scand. J. Plast. Reconstr. Surg. Suppl..

[7] Albrektsson T., Buser D., Sennerby L. (2012). Crestal bone loss and oral implants. Clin. Implant Dent. Relat. Res..

[8] Albrektsson T., Dahlin C., Jemt T., Sennerby L., Turri A., Wennerberg A. (2014). Is marginal bone loss around oral implants the result of a provoked foreign body reaction?. Clin. Implant Dent. Relat. Res..

[9] Degidi M., Artese L., Piattelli A., Scarano A., Shibli J.A., Piccirilli M., Perrotti V., Iezzi G. (2012). Histological and immunohistochemical evaluation of the peri-implant soft tissues around machined and acid-etched titanium healing abutments: A prospective randomised study. Clin. Oral Investig..

[10] Carcuac O., Berglundh T. (2014). Composition of Human Peri-implantitis and Periodontitis Lesions. J. Dent. Res..

[11] Lang N.P., Berglundh T. (2011). Periimplant diseases: Where are we now? Consensus of the Seventh European Workshop on periodontology. J. Clin. Periodontol..

[12] Becker S.T., Beck-Broichsitter B.E., Graetz C., Dörfer C.E., Wiltfang J., Häsler R. (2014). Peri-implantitis versus periodontitis: Functional differences indicated by transcriptome profiling. Clin. Implant Dent. Relat. Res..

[13] Koka S., Zarb G. (2012). On osseointegration: The healing adaptation principle in the context of osseosufficiency, osseoseparation, and dental implant failure. Int. J. Prosthodont..

[14] Qian J., Wennerberg A., Albrektsson T. (2012). Reasons for marginal bone loss around oral implants. Clin. Implant Dent. Relat. Res..

[15] Trindade R., Albrektsson T., Tengvall P., Wennerberg A. (2016). Foreign Body Reaction to Biomaterials: On Mechanisms for Buildup and Breakdown of Osseointegration. Clin. Implant Dent. Relat. Res..

[16] Albrektsson T., Becker W., Coli P., Jemt T., Mölne J., Sennerby L. (2019). Bone loss around oral and orthopedic implants: An immunologically based condition. Clin. Implant Dent. Relat. Res..

[17] Berglundh T., Armitage G., Araujo M.G., Avila-Ortiz G., Blanco J., Camargo P.M., Chen S., Cochran D., Derks J., Figuero E. (2018). Peri-implant diseases and conditions: Consensus report of workgroup 4 of the 2017 World Workshop on the Classification of Periodontal and Peri-Implant Diseases and Conditions. J. Clin. Periodontol..

[18] Faggion C.M., Listl S., Tu Y.K. (2010). Assessment of endpoints in studies on peri-implantitis treatment—A systematic review. J. Dent..

[19] Echt D.S., Liebson P.R., Mitchell L.B., Peters R.W., Obias-Manno D., Barker A.H., Arensberg D., Baker A., Friedman L., Greene H.L. (1991). Mortality and Morbidity in Patients Receiving Encainide, Flecainide, or Placebo—The Cardiac Arrhythmia Suppression Trial. N. Engl. J. Med..

[20] Giannobile W.V., Lang N.P. (2016). Are Dental Implants a Panacea or Should We Better Strive to Save Teeth?. J. Dent. Res..

[21] Listgarten M.A., Mao R., Robinson P.J. (1976). Periodontal probing and the relationship of the probe tip to periodontal tissues. J. Periodontol..

[22] Armitage G.C., Svanberg G.K., Loë H. (1977). Microscopic evaluation of clinical measurements of connective tissue attachment levels. J. Clin. Periodontol..

[23] Spray J.R., Garnick J.J., Doles L.R., Klawitter J.J. (1978). Microscopic demonstration of the position of periodontal probes. J. Periodontol..

[24] Ericsson I., Lindhe J. (1993). Probing depth at implants and teeth. An experimental study in the dog. J. Clin. Periodontol..

[25] Lang N.P., Wetzel A.C., Stich H., Caffesse R.G. (1994). Histologic probe penetration in healthy and inflamed peri-implant tissues. Clin. Oral Implants Res..

[26] Lekholm U., Adell R., Lindhe J., Brånemark P.I., Eriksson B., Rockler B., Lindvall A.M., Yoneyama T. (1986). Marginal tissue reactions at osseointegrated titanium fixtures. (II) A cross-sectional retrospective study. Int. J. Oral Maxillofac. Surg..

[27] Dierens M., Vandeweghe S., Kisch J., Nilner K., De Bruyn H. (2012). Long-term follow-up of turned single implants placed in periodontally healthy patients after 16–22 years: Radiographic and peri-implant outcome. Clin. Oral Implants Res..

[28] Winitsky N., Olgart K., Jemt T., Smedberg J.I. (2018). A retro-prospective long-term follow-up of Brånemark single implants in the anterior maxilla in young adults. Part 1: Clinical and radiographic parameters. Clin. Implant Dent. Relat. Res..

[29] Berglundh T., Lindhe J., Ericsson I., Marinello C.P., Liljenberg B., Thomsen P. (1991). The soft tissue barrier at implants and teeth. Clin. Oral Implants Res..

[30] Choquet V., Hermans M., Adriaenssens P., Daelemans P., Tarnow D.P., Malevez C. (2001). Clinical and radiographic evaluation of the papilla level adjacent to single-tooth dental implants. A retrospective study in the maxillary anterior region. J. Periodontol..

[31] Serino G., Turri A., Lang N.P. (2013). Probing at implants with peri-implantitis and its relation to clinical peri-implant bone loss. Clin. Oral Implants Res..

[32] Kan J.Y., Rungcharassaeng K., Umezu K., Kois J.C. (2003). Dimensions of peri-implant mucosa: An evaluation of maxillary anterior single implants in humans. J. Periodontol..

[33] Bergenblock S., Andersson B., Fürst B., Jemt T. (2012). Long-term follow-up of CeraOne single-implant restorations: An 18-year follow-up study based on a prospective patient cohort. Clin. Implant Dent. Relat. Res..

[34] Schou S., Holmstrup P., Stoltze K., Hjørting-Hansen E., Kornman K.S. (1993). Ligature-induced marginal inflammation around osseointegrated implants and ankylosed teeth. Clin. Oral Implants Res..

[35] Schou S., Holmstrup P., Stoltze K., Hjørting-Hansen E., Fiehn NESkovgaard L.T. (2002). Probing around implants and teeth with healthy or inflamed peri-implant mucosa/gingiva. A histologic comparison in cynomolgus monkeys (Macaca fascicularis). Clin. Oral Implants Res..

[36] Weber H.P., Crohin C.C., Fiorellini J.P. (2000). A 5-year prospective clinical and radiographic study of non-submerged dental implants. Clin. Oral Implants Res..

[37] Giannopoulou C., Bernard J.P., Buser D., Carrel A., Belser U.C. (2003). Effect of intracrevicular restoration margins on peri-implant health: Clinical, biochemical, and microbiologic findings around esthetic implants up to 9 years. Int. J. Oral Maxillofac. Implants.

[38] Emecen-Huja P., Eubank T.D., Shapiro V., Yildiz V., Tatakis D.N., Leblebicioglu B. (2013). Peri-implant versus periodontal wound healing. J. Clin. Periodontol..

[39] Fransson C., Wennström J., Berglundh T. (2008). Clinical characteristics and implant with a history of progressive bone loss. Clin. Oral Implants Res..

[40] Roos-Jansåker A.M., Lindahl C., Renvert H., Renvert S. (2006). Nine to fourteen-year follow-up of implant treatment. Part II: Presence of peri-implant lesions. J. Clin. Periodontol..

[41] French D., Cochran D.L., Ofec R. (2016). Retrospective cohort study of 4591 Straumann implants placed in 2060 patients in private practice with up to 10-year follow-up: The relationship between crestal bone level and soft tissue condition. Int. J. Oral Maxillofac. Implants.

[42] Broggini N., McManus L.M., Hermann J.S., Medina R.U., Oates T.W., Schenk R.K., Buser D., Mellonig J.T., Cochran D.L. (2003). Persistent acute inflammation at the implant-abutment interface. J. Dent. Res..

[43] Broggini N., McManus L.M., Hermann J.S., Medina R., Schenk R.K., Buser D., Cochran D.L. (2006). Peri-implant inflammation defined by the implant-abutment interface. J. Dent. Res..

[44] Buser D., Weber H.P., Donath K., Fiorellini J.P., Paquette D.W., Williams R.C. (1992). Soft tissue reactions to non-submerged unloaded titanium implant in beagle dogs. J. Periodontol..

[45] Sanz-Martín I., Sanz-Sánchez I., Carrillo de Albornoz A., Figuero E., Sanz M. (2018). Effects of modified abutment characteristics on peri-implant soft tissue health: A systematic review and meta-analysis. Clin. Oral Implants Res..

[46] Farina R., Filippi M., Brazzioli J., Tomasi C., Trombelli L. (2017). Bleeding on probing around dental implants: A retrospective study of associated factors. J. Clin. Periodontol..

[47] Jepsen S., Rühling A., Jepsen K., Ohlenbusch B., Albers H.K. (1996). Progressive peri-implantitis. Incidence and prediction of peri-implant attachment loss. Clin. Oral Implants Res..

[48] Monje A., Caballé-Serrano J., Nart J., Peñarrocha D., Wang H.L., Rakic M. (2018). Diagnostic accuracy of clinical parameters to monitor peri-implant conditions: A matched case-control study. J. Periodontol..

[49] Hashim D., Cionca N., Combescure C., Mombelli A. (2018). The diagnosis of peri-implantitis: A systematic review on the predictive value of bleeding on probing. Clin. Oral Implants Res..

[50] Doornewaard R., Jacquet W., Cosyn J., De Bruyn H. (2018). How do peri-implant biologic parameters correspond with implant survival and peri-implantitis? A critical review. Clin. Oral Implants Res..

[51] Attard N.J., Zarb G.A. (2004). Long-term treatment outcomes in edentulous patients with implant-fixed prostheses: The Toronto study. Int. J. Prosthodont..

[52] Astrand P., Ahlqvist J., Gunne J., Nilson H. (2008). Implant treatment of patients with edentulous jaws: A 20-year follow-up. Clin. Implant Dent. Relat. Res..

[53] Jemt T., Gyzander V., Britse A.Ö. (2015). Incidence of surgery related to problems with peri-implantitis: A retrospective study on patients followed up between 2003 and 2010 at one specialist clinic. Clin. Implant Dent. Relat. Res..

[54] Fransson C., Lekholm U., Jemt T., Berglundh T. (2005). Prevalence of subjects with progressive bone loss at implants. Clin. Oral Implants Res..

[55] Jemt T., Sundén Pikner S., Gröndahl K. (2015). Changes of marginal bone level in patients with progressive bone loss at Brånemark System® implants: A radiographic follow-up study over an average of 9 years. Clin. Implant Dent. Relat. Res..

[56] Renvert S., Lindahl C., Persson G.R. (2018). Occurrence of cases with peri-implant mucositis or peri-implantitis in a 21-26 year follow up study. J. Clin. Periodontol..

[57] Abrahamsson I., Berglundh T., Lindhe J. (1997). The mucosal barrier following abutment dis/reconnection. An experimental study in dogs. J. Clin. Periodontol..

[58] Rodríguez X., Vela X., Méndez V., Segalà M., Calvo-Guirado J.L., Tarnow D.P. (2013). The effect of abutment dis/reconnections on peri-implant bone resorption: A radiologic study of platform-switched and non-platform-switched implants placed in animals. Clin. Oral Implants Res..

[59] Koutouzis T., Gholami F., Reynolds J., Lundgren T., Kotsakis G.A. (2017). Abutment Disconnection/Reconnection Affects Peri-implant Marginal Bone Levels: A Meta-Analysis. Int. J. Oral Maxillofac. Implants.

[60] Wang Q.Q., Dai R., Cao C.Y., Fang H., Han M., Li Q.L. (2017). One-time versus repeated abutment connection for platform-switched implant: A systematic review and meta-analysis. PLoS ONE.

[61] Tallarico M., Caneva M., Meloni S.M., Xhanari E., Covani U., Canullo L. (2018). Definitive Abutments Placed at Implant Insertion and Never Removed: Is It an Effective Approach? A Systematic Review and Meta-Analysis of Randomized Controlled Trials. J. Oral Maxillofac. Surg..

[62] Lang N.P., Adler R., Joss A., Nyman S. (1990). Absence of bleeding on probing. An indicator of periodontal stability. J. Clin. Periodontol..

[63] Louropoulou A., Slot D.E., van der Weijden F.A. (2012). Titanium surface alterations following the use of different mechanical instruments: A systematic review. Clin. Oral Implants Res..

[64] Ruhling A., Kocher T., Kreusch J., Plagmann H.C. (1994). Treatment of subgingival implant surfaces with TeflonR-coated sonic and ultrasonic scaler tips and various implant curettes. An in vitro study. Clin. Oral Implant Res..

[65] Hallmon W.W., Waldrop T.C., Meffert R.M., Wade B.W. (1996). A comparative study of the effects of metallic, nonmetallic, and sonic instrumentation on titanium abutment surfaces. Int. J. Oral Maxillofac. Implants.

[66] Homiak A.W., Cook P.A., DeBoer J. (1992). Effect of hygiene instrumentation on titanium abutments: A scanning electron microscopy study. J. Prosthet. Dent..

[67] Cross-Poline G.N., Shaklee R.L., Stach D.J. (1997). Effect of implant curettes on titanium implant surfaces. Am. J. Dent..

[68] Souza P.P., Lerner U.H. (2013). The role of cytokines in inflammatory bone loss. Immunol. Investig..

[69] Noronha Oliveira M., Schunemann W.V.H., Mathew M.T., Henriques B., Magini R.S., Teughels W., Souza J.C.M. (2018). Can degradation products released from dental implants affect peri-implant tissues?. J. Periodontal Res..

[70] Suarez-Lopez del Amo F., Rudek I.E., Wagner V.P., Martins M.D., O’Valle F., Galindo-Moreno P., Giannobile W.V., Wang H.L., Castilho R.M. (2017). Titanium activates the DNA damage response pathway in oral epithelial cells: A pilot study. Int. J. Oral Maxillofac. Implants.

[71] Eger M., Sterer N., Liron T., Kohavi D., Gabet Y. (2017). Scaling of titanium implants entrains inflammation-induced osteolysis. Sci. Rep..

[72] Pettersson M., Kelk P., Belibasakis G.N., Bylund D., Molin Thoren M., Johansson A. (2017). Titanium ions form particles that activate and execute interleukin-1beta release from lipopolysaccharide-primed macrophages. J. Periodontal Res..

[73] Dodo C.G., Meirelles L., Aviles-Reyes A., Ruiz K.G.S., Abranches J., Cury A.A.D.B. (2017). Pro-inflammatory Analysis of Macrophages in Contact with Titanium Particles and Porphyromonas gingivalis. Braz. Dent. J..

[74] Wilson T.G., Valderrama P., Burbano M., Blansett J., Levine R., Kessler H., Rodrigues D.C. (2015). Foreign bodies associated with peri-implantitis human biopsies. J. Periodontol..

[75] Fretwurst T., Buzanich G., Nahles S., Woelber J.P., Riesemeier H., Nelson K. (2016). Metal elements in tissue with dental peri-implantitis: A pilot study. Clin. Oral Implants Res..

[76] Pettersson M., Pettersson J., Johansson A., Molin Thorén M. (2019). Titanium release in peri-implantitis. J. Oral Rehabil..

[77] Safioti L.M., Kotsakis G.A., Pozhitkov A.E., Chung W.O., Daubert D.M. (2017). Increased Levels of Dissolved Titanium Are Associated with Peri-Implantitis. A Cross-Sectional Study. J. Periodontol..

[78] Mombelli A., Hashim D., Cionca N. (2018). What is the impact of titanium particles and biocorrosion on implant survival and complications? A critical review. Clin. Oral Implants Res..

[79] Eger M., Hiram-Bab S., Liron T., Sterer N., Carmi Y., Kohavi D., Gabet Y. (2018). Mechanism and Prevention of Titanium Particle-Induced Inflammation and Osteolysis. Front. Immunol..

[80] Wang X., Li Y., Feng Y., Cheng H., Li D. (2019). Macrophage polarization in aseptic bone resorption around dental implants induced by Ti particles in a murine model. J. Periodontal Res..

[81] Purdue P.E., Koulouvaris P., Potter H.G., Nestor B.J., Sculco T.P. (2007). The cellular and molecular biology of periprosthetic osteolysis. Clin. Orthop. Relat. Res..

